# Resolvin D4 stereoassignment and its novel actions in host protection and bacterial clearance

**DOI:** 10.1038/srep18972

**Published:** 2016-01-08

**Authors:** Jeremy W. Winkler, Sarah K. Orr, Jesmond Dalli, Chien-Yee C. Cheng, Julia M. Sanger, Nan Chiang, Nicos A. Petasis, Charles N. Serhan

**Affiliations:** 1 Center for Experimental Therapeutics and Reperfusion Injury, Department of Anesthesiology, Perioperative and Pain Medicine, Harvard Institutes of Medicine, Brigham and Women’s Hospital and Harvard Medical School, Boston, Massachusetts 02115 USA; 2 Department of Chemistry and Loker Hydrocarbon Research Institute, University of Southern California, Los Angeles, California 90089 USA

## Abstract

Resolvins of the D-series are specialized pro-resolving lipid mediators that regulate cellular response by orchestrating resolution networks involved in host responses to injury and infection. Here, endogenous resolvin D4 was identified in human tissues and found to persist late into the resolution phase of acute murine *Staphylococcus aureus* infections. Completion of the first total synthesis of resolvin D4 established the absolute stereochemical configuration of RvD4 confirmed by matching with endogenous RvD4 from resolving exudates in dorsal pouch *S. aureus* infections. *In vivo*, RvD4 (ng/mouse) reduced neutrophilic infiltration (~40%) and enhanced uptake of apoptotic PMN (51%) by human dermal fibroblasts at concentrations as low as 0.1 nM. These results establish the complete stereochemistry of RvD4 as 4*S*,5*R*,17*S*-trihydroxydocosa-6*E*,8*E*,10*Z*,13*Z*,15*E*,19*Z*-hexaenoic acid and its novel pro-resolving actions in *S. aureus* infections as well as its potent ability to stimulate clearance of apoptotic cells by skin fibroblasts.

Resolution of acute inflammation involves local-acting chemical mediators that promote cellular trafficking and regulate their function^1^. Lipid mediators play key roles in regulating cytokine and chemokine production orchestrating the host response to injury and infection^2^,^3^. The resolution phase lipid mediators, in addition to regulating leukocytic infiltration, also promote clearance of apoptotic cells, debris and bacteria (reviewed in ref. 1). Chronic inflammation is now thought to arise from a potential failure to resolve local inflammation and is linked with several conditions including rheumatoid arthritis, cardiovascular disease and infection^1^,^4^. Resolution mediator networks actively shut down these pathways and regulate pro-resolving pathways that may in theory allow for disease prevention via resolution pharmacology and therapeutic strategies.

Docosahexaenoic acid (DHA) is enriched in the brain, skin, central nervous system and eye. Appropriate consumption of DHA is linked with several wide-ranging effects on health and disease^5^. Towards appreciating the mechanisms underlying DHA impact, a family of DHA-derived local mediators termed D-series resolvins with unique biological activity and temporal profiles were identified during the resolution phase of acute inflammation. This family of autacoids is positioned at the nexus between a host’s early response to inflammatory challenge and resolution pathways where the beginning or initiating programs signal the end or termination^1^. Establishing the complete stereochemistry of each specific SPM has permitted confirmation of their potent actions as well as determination of their novel specific biological actions including organ protection, tissue remodeling and microbial containment^1^,^6^. From the resolvin (resolution phase interaction products) D series, the absolute stereochemistries of RvD1 (7*S*,8*R*,17*S*-trihydroxydocosa-4*Z*,9*E*,11*E*,13*Z*,15*E*,19*Z*-hexaenoic acid), RvD2 (7*S*,16*R*,17*S*-trihydroxydocosa-4*Z*,8*E*,10*Z*,12*E*,14*E*,19*Z*-hexaenoic acid), and recently RvD3 (4*S*,11*R*,17*S*-trihydroxydocosa-5*Z*,7*E*,9*E*,13*Z*,15*E*,19*Z*-hexaenoic acid), were each established along with select potent biological actions (for example, see ref. 7). As originally described as the biological isolate, RvD4 possesses potent biological actions that include reducing neutrophilic infiltration, inhibiting glial cell cytokine production and regulating leukocyte diapedesis at concentrations as low as 1 nM^8^. Biologically isolated RvD4 also potently reduces recruitment of neutrophils in murine peritonitis and dorsal skin pouch in response to either opsonized zymosan or TNF-α^9^. In addition, RvD4 is also produced during ischemic kidney injury and exerts organ protection^10^.

Given the potent biological actions described for endogenous RvD4, the unambiguous stereochemistry and complete structure of the bioactive RvD4 remained to be established^8^. The structure of RvD4 was originally deduced based on biosynthetic evidence as well as liquid chromatography-tandem mass spectrometry (LC-MS-MS) and GC-MS metabololipidomic profiling of the bioactive compound from exudates and human leukocytes, which did not allow for establishing the double-bond triene geometry as well as the chirality of the alcohol at the 5 position. In the present report using material obtained by a total organic synthesis approach, we establish the complete stereochemistry of RvD4 as 4*S*,5*R*,17*S*-trihydroxydocosa-6*E*,8*E*,10*Z*,13*Z*,15*E*,19*Z*-hexaenoic acid, confirm its potent biological actions and establish its new specific pro-resolving action in dermal inflammation and infection.

## Results

### RvD4 is produced in bioactive levels during *S. aureus* infection

**Figure 1:**
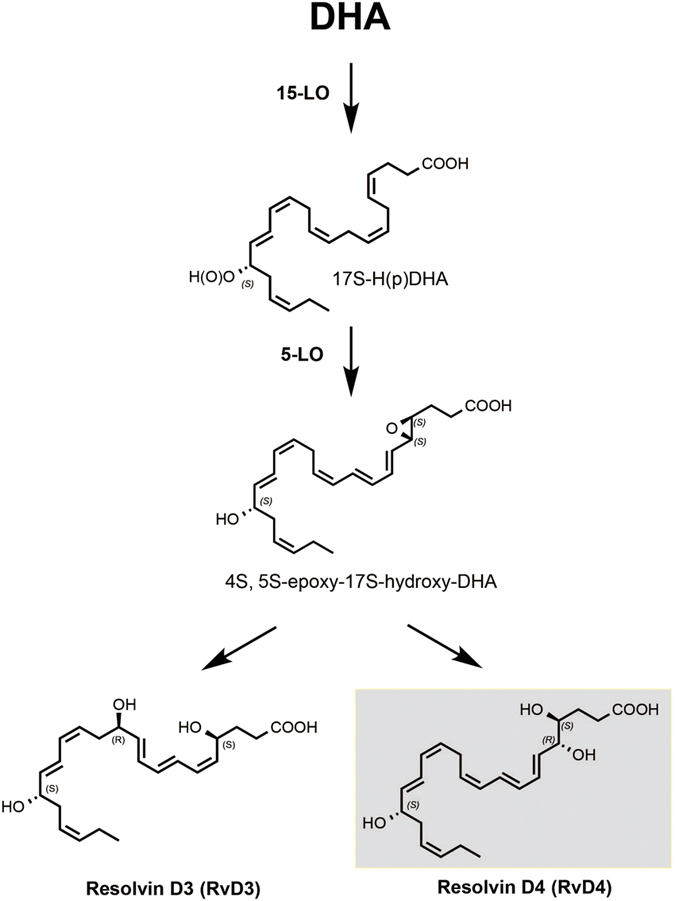
Biosynthetic pathway of Resolvin D4 and Resolvin D3. RvD4 is formed *via* a proposed 4,5-epoxide intermediate^8^ in a stereo-controlled manner confirmed by matching with synthetic compound. Enzymatic hydrolysis at the C-5 position to open the epoxide produces the 4*S*,5*R* hydroxy-containing RvD3, or enzymatic attack at the C-11 position affords the 4*S*,11*R* hydroxy-containing RvD4.

**Figure 2:**
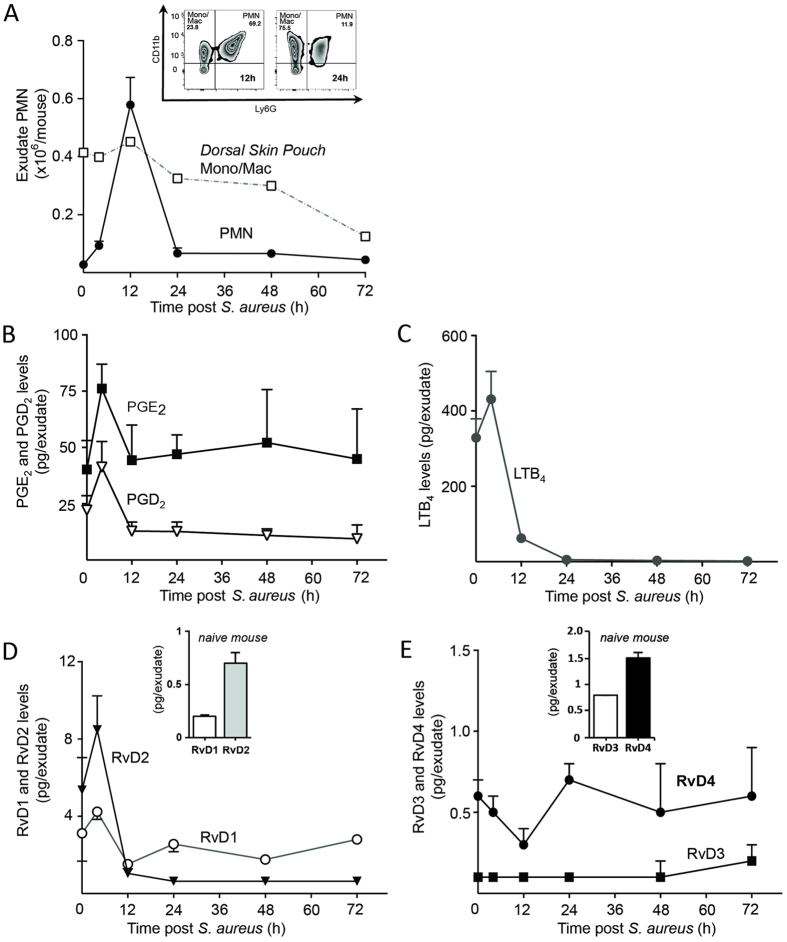
Endogenous production of RvD4 in *S. aureus*-initiated skin infection. Lipid mediator levels were assessed by LC-MS-MS-based LM metabololipidomics following solid phase extraction of dorsal pouch infectious exudates. (**A**) Time course of neutrophil recruitment into the dorsal pouch inoculated with *S. aureus* (10^5^ c.f.u.) at time 0 and exudates collected at indicated intervals. Total cell counts were enumerated using light microscopy, and monocytes and PMN were identified using flow cytometry (inset). (**B**,**C**) Exudate levels of prostaglandins and leukotrienes, (**D**,**E**) D-series Resolvin levels. Results are mean ± SEM, n = at least 3 separate experiments.

To identify relevant SPMs with protective actions during the resolution programme we used a well-established dorsal pouch model for murine *S. aureus* skin infection^6^ with focus on the endogenous formation and appearance of Resolvin D4 (Fig. 1). Inoculation of *S. aureus* at 10^5^ c.f.u. gave a self-limited inflammatory response with polymorphonuclear leukocyte (PMN) numbers reaching a maximum at 12 hr (T_max_), followed by a sharp decline and return to basal levels by 24 hr. At 12 hr, monocytes/macrophages gave a more persistent presence and remained elevated throughout the resolution phase into the later time points (Fig. 2A), consistent with their role in clearance of apoptotic PMN and cellular debris^8^,^11^.

In this time course, the LM and SPM profiles from infectious exudates were monitored using LC-MS-MS-based lipid mediator metabololipidomics. The inflammation initiating prostaglandins and leukotrienes LTB_4_, PGE_2_ and PGD_2_ increased sharply in response to a bacterial inoculation followed by a return to basal levels (Fig. 2B,C). In these infectious purulent exudates we normalized the recovery based on individual lavage volume to determine the temporal profile of the D-series resolvins and found them present in low picomolar concentrations. RvD1 and RvD2 reached a maximum presence at 12 hr. RvD3 was observed at low levels throughout the time course of self-limited infection whereas RvD4 gave a unique pattern with its production persisting late into the resolution phase. These results suggest that RvD4 is constituently present and is differentially regulated and produced to regulate processes in the resolution of infection (Fig. 2D,E).

### Total organic synthesis of RvD4

**Figure 3:**
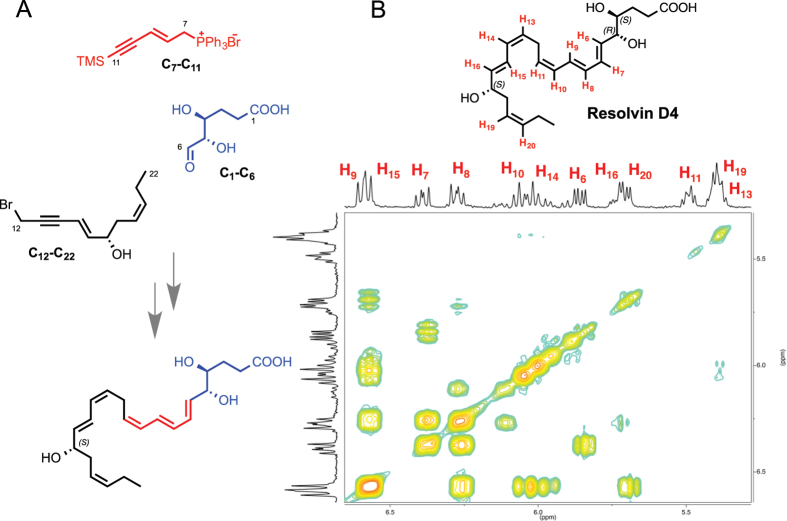
Total synthesis and characterization of Resolvin D4. Synthetic RvD4 was accomplished by total organic synthesis from chirally pure starting materials prepared by multi-step total organic synthesis and characterized using NMR spectroscopy. (**A**) Synthetic precursors were prepared from enantiomerically pure commercially available starting materials and coupled using carbon-carbon bond coupling reactions between precursor C_1_–C_6_ in (blue), C_7_–C_11_ (in red), and C_12_–C_22_ (in black) to ensure absolute regio- and stereochemical assignment; (**B**) Double-bond geometry was assigned using 2-dimensional ^1^H-^1^H NMR using a Varian NMRS 600 MHz NMR spectrometer at 25 °C on a 5 mm Triple Resonance PFG^1^H probe and referenced to the CD_3_OD internal standard. The rainbow plot depicts positive contours of cross peaks along the diagonal axis allowing for the full and detailed proton assignment. The zoomed-in region highlights the Z/E olefinic protons H_6_–H_11_, H_13_–H_16_ and H_19_–H_20_. This in addition to the full ^1^H-NMR spectrum of chemical shifts and coupling constants confirmed the structure of Resolvin D4.

To establish the complete stereochemistry for RvD4 and assign the double bond geometry around C7 to C11 and the chirality of the alcohol at the C5 position, we employed a total organic synthesis approach that was also used in establishing the complete stereochemistry for other SPM^12^. Along these lines the complete total synthesis of RvD4 was accomplished using a 22-step total organic synthetic scheme, to afford stereochemically pure 4*S*,5*R*,17*S*-trihydroxydocosa-6*E*,8*E*,10*Z*,13*Z*,15*E*,19*Z*-hexaenoic acid, RvD4, shown in Fig. 3A. The hydroxyl groups positioned at the 4*S*, 5*R* carbon positions proved similarly to be a problematic complication in which the ester precursor rearranged to form a mixture of the ester as well as the 5- and 6-membered lactone, as observed similarly with the synthesis of RvD3^12^.

With these aforementioned challenges in mind we began our synthesis with the ring opening of chirally pure d-erythrose to set the *4S,* 5*R* hydroxyl groups in the proposed stereoconfiguration. Next, we took advantage of a Wittig coupling to form the carbon-carbon bond at the 6-position between the Wittig salt and aldehyde and through additional manipulation built the conjugated triene system. The 17*S*-hydroxy position was set in place through a ring opening of chiral *R-*glycidol. The final carbon-carbon bond was formed through a copper-mediated cross coupling of an allylic-bromide with terminal alkyne C11 to afford the protected bis-acetylenic precursor in good yield. Because of the sensitive nature of each metabolite, RvD4 at this stage required careful handling. The bis-acetylenic precursor was reduced prior to the deprotection; however, Zn/Cu/Ag hydrogenation was too mild in the presence of bulky protecting groups. Therefore, Lindlar catalyst was employed and subsequent desilylation allowed for the synthesis of the final compound 4*S*,5*R*,17*S*-trihydroxydocosa-6*E*,8*E*,10*Z*,13*Z*,15*E*,19*Z*-hexaenoic acid without decomposition. The absolute structure was confirmed by 2D ^1^H-^1^H nuclear magnetic resonance (NMR) spectroscopy for full proton assignment (Fig. 3B).

### Matching of endogenous RvD4 with synthetic materials

**Figure 4:**
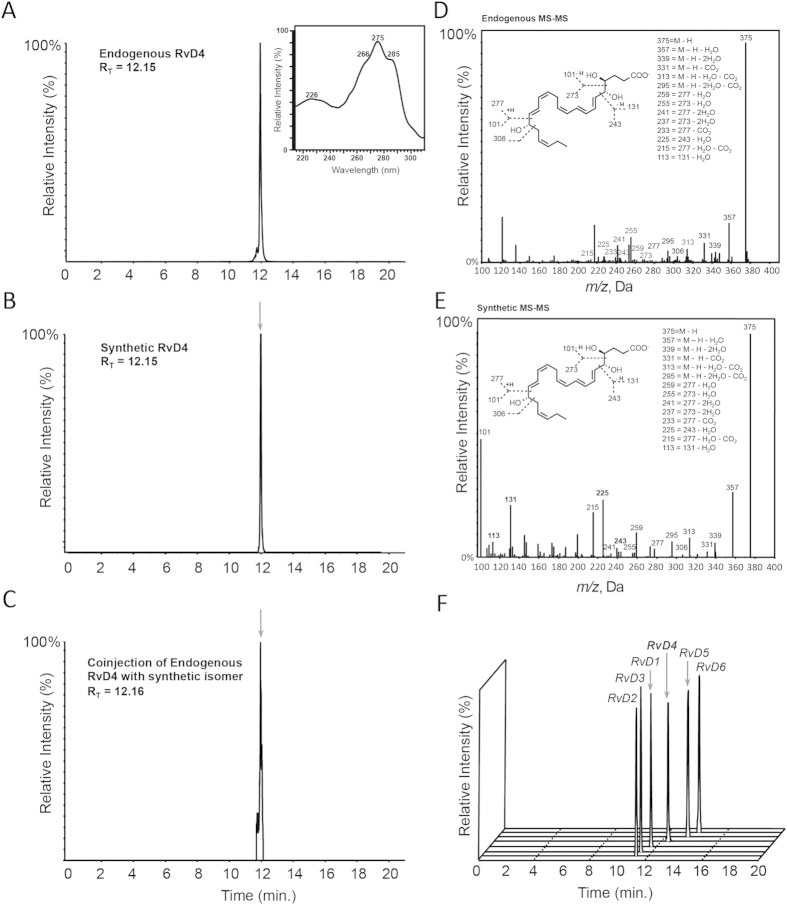
Matching of endogenous RvD4 with synthetic compound. Endogenous RvD4 was obtained from murine dorsal pouches inoculated with *S. aureus* (10^5^ c.f.u.) and collected at 4 hr. Selected ion MRM chromatograms (m/z 375-101) of (**A**) endogenous resolving exudate-derived RvD4 and characteristic UV-absorption spectra (inset) and (**B**) synthetic isomer mediator; (**C**) LC-chromatograms for coinjection of endogenous RvD4 and synthetic material; (**D**,**E**) Representative MS-MS spectra for endogenous and synthetic RvD4; (**F**) Select ion MRM chromatograms for the D-series resolvins from infectious exudates. Results are representative of n = 3.

We next matched the physical properties of the material obtained by total organic synthesis to that of endogenous RvD4. Our first criterion for the matching of authentic material with synthetic compound was by retention time. The chromatographic signature of both the endogenous and synthetic material demonstrated identical retention profiles by reverse phase high pressure liquid chromatography (HPLC) with a sharp peak eluting with retention time (T_R_) of 12.2 min (Fig. 4A,B). Coinjection of the authentic RvD4 isolated from purulent inflammatory exudates with synthetic compound confirmed that these products co-eluted (Fig. 4C).

Further validation of synthetic material was confirmed by assessing the UV absorbance profile and tandem mass spectrometry (MS-MS) patterns. The synthetic material displayed UV absorbance profile with a triplet band of absorption, 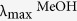, at 275 nm and shoulders at 266 and 285 that is characteristic of a triene conjugated double-bond system^1^. The synthetic material also gave a broad shoulder with absorption 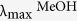 at ~226 nm (Fig. 4A inset) that is characteristic of a conjugated double-bond system. This absorbance profile was in accord with published values for endogenous RvD4^8^. We next obtained MS-MS fragmentation pattern using an LC-MS-MS metabololipidomics-based approach as in ref. 13, demonstrating that the synthetic and endogenous RvD4 matched in physical properties. The MS-MS profile for endogenous RvD4 as well as its synthetic material gave a parent ion with *m/z* 375 = M-H and daughter ions with *m/z* 357 = M-H-H_2_O, *m/z* 339 = M-H-2H_2_O, *m/z* 331 = M-H-CO_2_, *m/z* 313 = M-H-H_2_O-CO_2_, *m/z* 295 = M-H-2H_2_O-CO_2_, *m/z* 259 = 277-H_2_O, *m/z* 255 = 273-H_2_O, *m/z* 241 = 277 -2H_2_O, *m/z* 237 = 273-2H_2_O, *m/z* 233 = 277-CO_2_, *m/z* 225 = 243-H_2_O, *m/z* 215 = 277-H_2_O-CO_2_, *m/z* 113 = 131-H_2_O (Fig. 4D,E). To place RvD4 within the D-series resolvins we profiled the infectious exudates and found that RvD4 eluted last of the trihydroxyl resolvin family giving it the most lipophilic characteristic of the four (Fig. 4F).

### RvD4 stops neutrophil infiltration in sterile peritonitis and *S. aureus* skin infection

**Figure 5:**
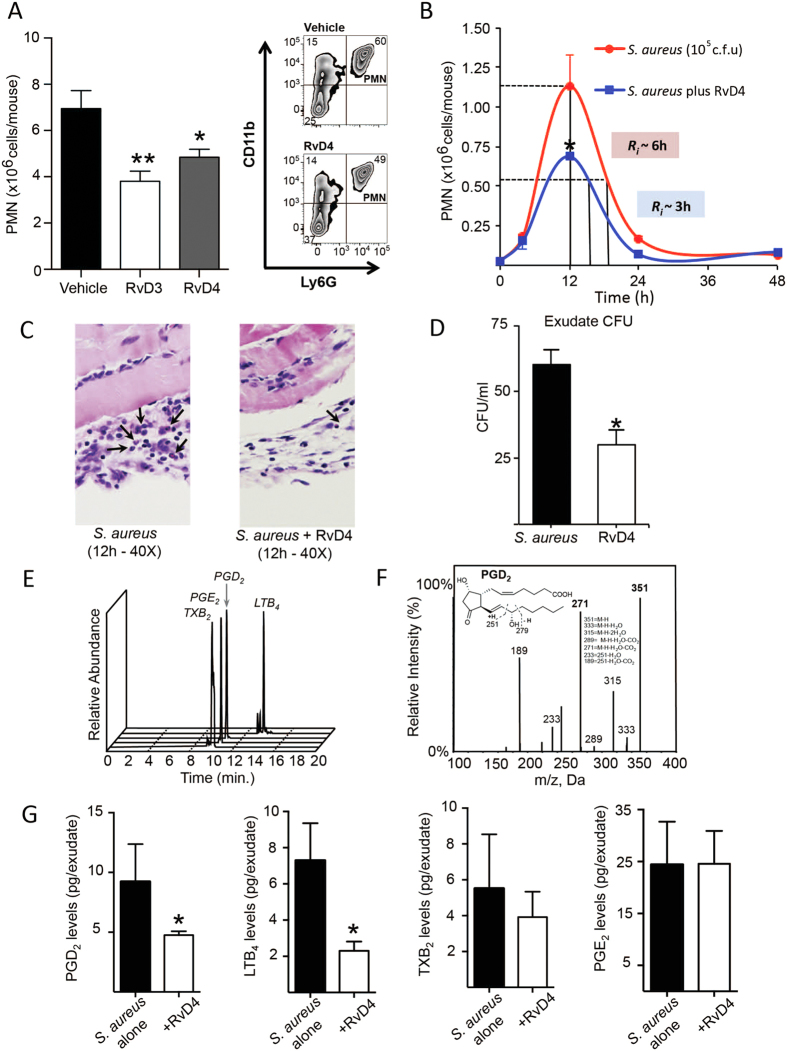
RvD4 reduces neutrophil infiltration in zymosan-initiated peritonitis and *S. aureus* infection. 6–8-week-old male FVB mice were injected *i.p*. with 10 ng RvD3, RvD4, or vehicle (saline containing 0.01% EtOH) along with 1 mg zymosan A. Exudates were collected 4 hr later. (**A**) Total cell counts were enumerated using light microscopy, and PMN were identified using flow cytometry. Results are mean ± SEM, n = 3–4 mice. Statistical analysis was performed using two-way ANOVA. *p < 0.05, **p < 0.01, vs. zymosan A alone. Murine dorsal pouches were inoculated with live *S. aureus* (10^5^ c.f.u.) followed by injection of vehicle or RvD4 (200 ng/mouse); exudates were collected at 4, 12, 24, and 48 hr; (**B**) Exudate PMN numbers and resolution indices were calculated; (**C**) H & E staining of airpouch lining (arrows denote PMN; (**D**) Bacterial counts were expressed as colony forming unit (c.f.u.); (**E–G**) Endogenous eicosanoids in infectious exudates from mice given vehicle or RvD4 (200ng/mouse); (**E**) MRM chromatogram for exudate eicosanoids; (**F**) Representative MS-MS spectra employed for the identification of PGD_2_; (**G**) Exudate prostanoid levels. Results are mean ± SEM, n = 3 separate experiments. Statistical analysis was performed using Student’s *t*-test. *p < 0.05, vs. *S. aureus* alone.

We next sought to confirm the potent anti-inflammatory and pro-resolving actions of RvD4 with synthetic material. First we assessed its actions in regulating the acute inflammatory response initiated by zymosan A in a peritonitis model. Zymosan administration *via i.p*. injection gave rapid PMN infiltration into the peritoneal cavity at 4 hr. Shown on the representative dot plots and bar graphs, RvD4 at 10 ng/mouse decreased percent of PMN, giving ~31% reduction at 4 hours (Fig. 5A). For comparison, administration of an equal amount of the potent immunoresolvent RvD3^7^ also gave ~44% reduction in PMN infiltration. These results confirm the potent pro-resolving anti-inflammatory properties initially described for the bioactive RvD4^8^ with synthetic material.

With the biological activity of RvD4 confirmed, we next assessed its *in vivo* actions in *S. aureus-*mediated infection probing its action as well as temporal formation. To determine its role in bacterial killing and host protection during *S. aureus* infections, RvD4 was administered at 200 ng/mouse concomitantly with a self-limited bacterial inoculation (10^5^ c.f.u) into the dorsal pouch of male FVB mice. Resolution indices were then measured. RvD4 potently reduced neutrophilic infiltration by ~40% at T_max_ (12 h) and shortened the resolution interval in mice treated with RvD4 from 6 to 3 hr (Fig. 5B). Analysis by histochemical staining of dorsal pouch lining from mice administered vehicle or RvD4 gave lower PMN levels in the cavity in mice treated with RvD4 (Fig. 5C). Treatment with RvD4 also significantly lowered bacterial counts when compared to vehicle control (Fig. 5D). In addition, RvD4 enhanced the phagocytosis of *S. aureus* by isolated mouse macrophages by ~40% at 10nM (data not shown). RvD4 also regulated inflammation-initiating eicosanoids in the inflammatory exudates including LTB_4_ and PGD_2_ by 68 and 48% respectively, demonstrating potent anti-inflammatory actions *in vivo* (Fig. 5E–G).

### RvD4 exerts pro-resolving actions with human neutrophils and macrophages

**Figure 6:**
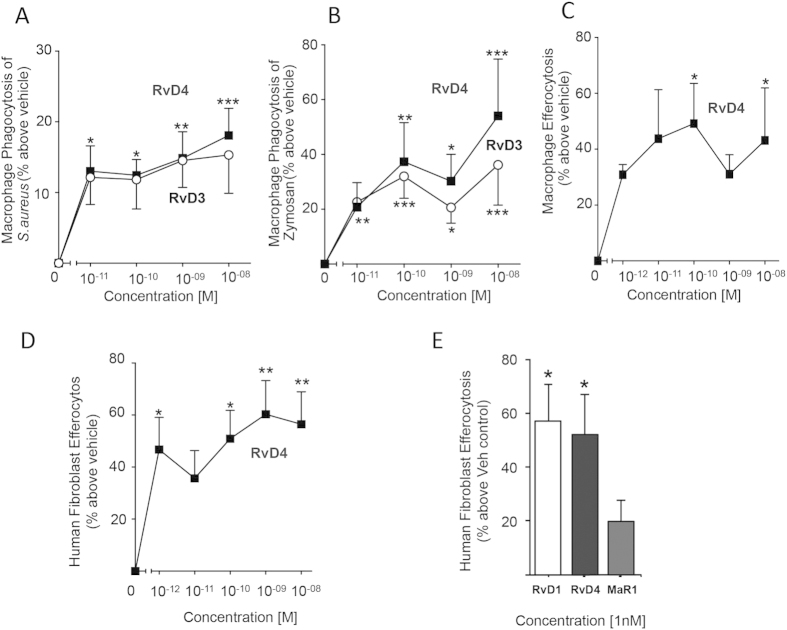
RvD4 enhances human macrophage and dermal fibroblast phagocytosis and efferocytosis. Isolated human macrophage were incubated with vehicle or RvD4 (10 pM-10 nM; 15 min, 37 °C), and then with (**A**) fluorescently labeled *S. aureus* (50:1); (**B**) FITC labeled opsonized zymosan A (10:1); or (**C**) fluorescent labeled apoptotic neutrophil for 1 hr (3:1). Results are mean ± SEM, n = 3 healthy subjects. *p < 0.05, **p < 0.01, ***p < 0.001 compared to vehicle. (**D**) Human-derived dermal fibroblasts were incubated with vehicle or RvD4 (1 pM-10 nM; 15 min, 37 °C), then with apoptotic neutrophil (3:1) for 1 hr; (**E**) Rank order comparison. Fibroblasts were incubated with RvD1, RvD4 or MaR1 (1nM; 15 min, 37 °C) with apoptotic neutrophils for 1 h and efferocytosis assessed. Results are mean ± SEM, n = 3–4. *p < 0.05, **p < 0.01 compared to vehicle.

To further elucidate its host-protective actions, we sought to establish the potent anti-inflammatory and pro-resolving actions of RvD4 with isolated human leukocytes. RvD4 (0.01 nM to 10 nM) promoted bacterial phagocytosis by human macrophages (12–18%) to a similar extent as the pro-resolving mediator RvD3 (Fig. 6A). RvD4 also promoted the phagocytosis of opsonized zymosan A at concentrations as low as 0.1–10 nM, significantly enhancing macrophage uptake by 40–60% above vehicle controls (Fig. 6B).

The clearance of apoptotic PMN during inflammation is a cellular hallmark in resolution^1^,^14^. To determine RvD4’s role in the clearance of apoptotic neutrophils we assessed whether RvD4 promoted macrophage efferocytosis. RvD4 statistically significantly increased macrophage efferocytosis of apoptotic neutrophils by 40–50% at concentrations as low as 0.1–10 nM (Fig. 6C). These results suggest that RvD4 enhances human macrophage functions in clearing cellular debris and microbial particles. Together these results provide evidence that RvD4 is pro-resolving with human phagocytes.

### RvD4 exerts pro-resolving actions with human fibroblasts: Comparison with RvD1 and MaR1

Given that RvD4 displayed potent pro-resolving actions in skin infection, we investigated whether it also acts on human dermal fibroblasts. RvD4 potently promoted the clearance of apoptotic PMN by human fibroblasts (Fig. 6D) by ~59% at 1 nM. Of the SPM tested, RvD4 and RvD1 at 1 nM gave the highest increase in apoptotic cell uptake (~52 and 57%). These findings demonstrate new action of SPM such as RvD1 and MaR1 (7*R*,14*S*-dihydroxydocosa-4*Z*,8*E*,10*E*,12*Z*,16*Z*,19*Z*-hexaenoic acid) in resolution and that RvD4 displays potent actions with human dermal fibroblasts (Fig. 6E).

### RvD4 identification in tissue in human and marine organisms

Since SPMs are identified in marine organisms and human tissues, suggesting potential physiologic role(s) in these organisms^1^,^15^, we next assessed the presence of RvD4 in relation to other members of the D-series resolvins in human plasma, human serum^13^, human breast milk^16^ as well as murine spleen, brain and sardines (Table 1). The presence of endogenous RvD4 in these samples at picogram levels (10–100 pg) suggests a utility in the evolutionary conservation in structure function across species associated with its role in the resolution process.

## Discussion

In the present report we establish the complete stereochemical assignment of RvD4 as 4*S*,5*R*,17*S*-trihydroxydocosa-6*E*,8*E*,10*Z*,13*Z*,15*E*,19*Z*-hexaenoic acid by matching endogenous material isolated from purulent inflammatory exudates with synthetic compound (Fig. 4) made by total organic synthesis (Fig. 3). RvD4 was temporally regulated during *S. aureus* infection as well as remained at bioactive levels (~ pM) in the late stages of resolution (Fig. 2). Administration of RvD4 *in vivo* counter regulated inflammatory initiating eicosanoids in *S. aureus* infection and shortened resolution indices during infection (Fig. 5), concepts on which the edifice of resolution is based. Administration of RvD4 in both sterile inflammation and microbial infection gave a reduction in neutrophil recruitment *in vivo* (Figs 5 and 6). RvD4 also enhanced macrophage responses with human macrophages and enhanced fibroblast responses with human fibroblasts in efferocytosis of apoptotic PMN and sterile zymosan (Fig. 6). Furthermore RvD4 was found to be present in a number of tissues across species suggesting its utility through evolutionary conservation (Table 1).

The biosynthesis of this family of compounds by lipoxygenase enzymes affords their unique structures and function. RvD4 and its congener RvD3 are formed *via* a common 4, 5-epoxide involving a second oxygenation pathway by lipoxygenase enzymes. The divergence between RvD3 and RvD4 occurs from the insertion of water by enzymatic hydrolysis at the C11 and C5 position through a carbocation intermediate to afford RvD3 and RvD4, respectively (Fig. 1). Both compounds have distinct behaviors based on their arrangement of chiral hydroxyl stereocenters and double-bond triene-diene configuration giving them each a distinct activity profile and set of physical characteristics such as absorbance and retention time. Synthetic RvD3 demonstrated greater potency in a mouse peritonitis model (Fig. 5) while synthetic RvD4 significantly increased efferocytosis in human fibroblasts by promoting the clearance of apoptotic PMN, while RvD3 was not significant (Fig. 6).

Resolvins can alleviate both the bacterial burden in a skin *S. aureus* infection as well as in lung infection models^6^,^17^. Resolvins are produced and act locally in a time-dependent fashion to intercede with an overbearing neutrophilic response that can lead to chronic inflammation, tissue fibrosis, and scarring. Antibiotic-resistant *S. aureus* skin infections are widespread and a leading cause of hospital-acquired infection^6^. The acute inflammatory response involves a number of inflammation-initiating chemical mediators including thromboxanes, prostaglandins and leukotrienes as well as cytokines and chemokines. The D-series resolvins arbitrate this interaction by shutting down proinflammatory pathways and turning on resolution networks^8^,^11^,^18^,^19^. In the *S. aureus* infection model, RvD1 and RvD2 levels traced with the eicosanoids followed by a temporally distinct response of Resolvin D3 and D4 at later time points, thus suggesting differential roles in the 7,8 vs. 4,5 epoxide pathway shared by RvD4 and RvD3 during late-stage clearance through phagocytosis of foreign bacteria and apoptotic PMN by macrophages and dermal fibroblasts (Fig. 6). Administration of RvD4 *in vivo* stopped leukocyte influx to the site of infection in the dorsal pouch cavity as well as the inflammation-initiating eicosanoids by reducing levels of PGD_2_ and LTB_4_.

Earlier work has shown that SPM actions are not limited to leukocytes but can stimulate endothelial cell-leukocyte interactions, suggesting a more prominent role in the resolution phase. Fibroblasts are known to be a critical component in wound healing and tissue remodeling for their restorative properties in supporting the structural framework for tissue and repair^20^,^21^. Lung fibroblasts and periodontal ligament fibroblasts have been shown to respond to SPMs under smoke-induced lung inflammation^22^ and periodontal disease^23^, respectively. Dermal fibroblasts were found to phagocytize apoptotic PMN and opsonized beads as previously reported in refs 24 and 25, and this action was enhanced by RvD4, providing a novel mechanism of action in the resolution of dermal infectious inflammation. The role SPM play in signaling dermal fibroblasts during resolution has not been investigated before. We investigated RvD4’s role in signaling dermal fibroblast clearance of apoptotic PMN and found RvD4 actively promotes phagocytosis at subnanomolar concentrations, suggesting it as a critical player in the resolution regiment of this potent class of autacoids.

In summation, the present findings report the complete stereochemistry of RvD4, endogenous production of RvD4 during infection, RvD4’s regulation of leukocyte recruitment in sterile inflammation and infection, counter-regulation of eicosanoids, phagocyte-directed action with human macrophages (defining its pro-resolving actions), increase of fibroblast phagocytosis as well as RvD4 identification across several species. These findings suggest RvD4 plays a pivotal role(s) in infectious resolution as well as gives insight into its temporal vignette in the endogenous production of these potent SPM-autacoids during resolution and return to homeostasis.

## Significance

These results establish RvD4 stereochemistry and role in host protection and resolution of inflammation during bacterial infection on specific target cell types including neutrophils and macrophages. RvD4 diminishes inflammation by promoting clearance of a bacterial burden and promotes the efferocytosis of apoptotic PMN. For the first time an SPM was shown to have a direct proresolving action on a new target cell type, human dermal fibroblasts, where RvD4 potently stimulates phagocytosis and efferocytosis of cellular debris and apoptotic neutrophils. The complete stereochemical assignment of RvD4 with its synthetic precursor was confirmed by matching both physical properties as well as biological actions to confirm the complete stereochemical assignment, which now opens a new path for therapeutic developments based on RvD4 structure and action. The identification of endogenous D-series resolvins and RvD4 displays a unique spatio-temporal ensemble and is produced at biologically relevant levels during *S. aureus-*initiated infection suggesting an endogenous host defense mechanism triggering resolution and host protection during infectious inflammation. These results from RvD4 provide structural evidence for a new approach to host-directed therapies that now take into account the role of fibroblasts in resolution as well as infection.

## Methods

### Materials

RPMI 1640 and DPBS (with or without calcium and magnesium) were purchased from Lonza (Hopkinton, MA, USA). Ficoll-Histopaque 1077-1, lipopolysaccharide (LPS) and zymosan A were obtained from Sigma-Aldrich (St. Louis, MO, USA). Human recombinant granulocyte-monocyte colony stimulating factor (GM-CSF), macrophage colony-stimulating factor, interferon γ (IFN-γ) and interleukin-4 were purchased from R&D Systems (Minneapolis, MN, USA). Fetal calf sera, ampicillin and bac-to-bac baculovirus expression system were from Invitrogen (Grand Island, NY, USA). FITC rat anti-mouse Ly6G (clone IAS) and purified rat anti-mouse CD16/32 (mouse BD Fc block) were purchased from BD Biosciences (San Jose, CA), and PE rat anti-mouse F4/80 (clone BM8) and PerCP/Cy5.5 rat anti-mouse CD11b (clone Mac-1) were purchased from eBioscience (San Diego, CA). C18 SPE columns were from Waters (Milford, MA, USA). Poroshell C18 columns were from Phenomenex; fluorescently conjugated zymosan A was from Invitrogen. All liquid chromatography solvents were from Fisher Scientific (Pittsburgh, PA, USA). DHA along with d_4_-leukotriene B_4_ (d_4_-LTB_4_), d_5_-lipoxin A_4_ (d_5_-LXA_4_), d_4_-prostaglandin E_2_ (d_4_-PGE_2_), d_8_-5S-HETE and d_5_-Resolvin D2 (d_5_-RvD2) were purchased from Cayman Chemicals (Ann Arbor, MI, USA). Fresh human leukocytes were prepared from peripheral blood of deidentified and consenting healthy volunteers. Informed consent was obtained from all subjects. The protocol was approved by the Partners Human Research Committee (Protocol number 1999P001297) and was in accordance with the Declaration of Helsinki.

### Bacteria growth

*S. aureus* were cultured in LB broth and harvested at mid-log phase (OD_600_ ~ 0.5, 5 × 10^8^ c.f.u./ml) and washed in sterile saline before inoculation into mouse dorsal pouch.

### NMR of Synthetic RvD4

The proton chemical shift and coupling constants for RvD4 were recorded as follows (see Fig. 2 for further details). ^1^H NMR (600 MHz, MeOD) δ 6.51–6.43 (dd, *J* = 15.0, 11.1 Hz, 2H), 6.27 (dd, *J* = 15.3, 10.7 Hz, 1H), 6.15 (dd, *J* = 14.7, 10.7 Hz, 1H), 5.94 (t, *J* = 10.9 Hz, 1H), 5.90 (t, *J* = 10.9 Hz, 1H), 5.74 (dd, *J* = 15.2, 6.8 Hz, 1H), 5.59 (dd, *J* = 15.0, 6.6 Hz, 2H), 5.41–5.34 (m, 1H), 5.33–5.23 (m, 3H), 4.04 (q, *J* = 6.5 Hz, 1H), 3.89 (t, *J* = 6.0 Hz, 1H), 3.45–3.39 (m, 1H), 3.00 (t, *J* = 7.7 Hz, 2H), 2.32–2.13 (m, 4H), 1.95 (q, *J* = 7.6 Hz, 2H), 1.75 (ddd, *J* = 14.7, 7.1, 3.2 Hz, 1H), 1.58 (dq, *J* = 15.1, 7.5 Hz, 1H), 0.86 (t, *J* = 7.5 Hz, 3H).

### Acute Inflammation

*S. aureus* (10^5^ c.f.u./mouse) microbial infection was initiated in dorsal pouch of mice (6 to 8 weeks-old FVB mice) purchased from Charles River Laboratories and fed Laboratory Rodent Diet 20–5058 (Lab Diet, Purina Mills). Experiments were approved by the Standing Committee on Animals of Harvard Medical School (protocol no. 02570) and complied with institutional and U.S. National Institutes of Health (NIH) guidelines.

#### Dorsal skin pouch

Mice were anesthetized with isoflurane, and 3.0 ml of sterile air was injected subcutaneously on days 0 and 3 between the shoulders and into the back of male mice. On day 6, *S. aureus* (10^5^ c.f.u./mouse) was administered by intra-pouch injection in combination with either RvD4 (200 ng/mouse) or sterile saline. No apparent changes were observed in animal behavior, and mice were euthanized and lavages were collected at designated time points (4, 12, 24, 36, 48 and 72 hr) with 3 mL DPBS^−/−^. Leukocytes were enumerated and differential counts conducted by flow cytometry and graphed by curve smoothing. Aliquots of lavage were serial diluted and plated on agar plates and cultured overnight at 37 °C to determine bacterial counts. Skin punch biopsies were collected and stained for hematoxylin-eosin staining.

#### Murine peritonitis

1 mg/ml of zymosan was injected i.p. in parallel with RvD3 (10 ng), RvD4 (10 ng), or vehicle in 100 μl saline. At 4 h, peritoneal lavages were collected and cell counts enumerated by flow cytometry.

### Flow Cytometry

Air pouch exudates were lavaged and analyzed following previous procedure outlined in ref. 7. Cells were suspended in FACS buffer (5% BSA in DPBS) and incubated for 40 min at 4 °C with individual antibody or combinations of FITC-conjugated anti-mouse LY6G (clone 1AS) for PMN and PE-conjugated anti-mouse F4/80 Ab (clone BM8) for MΦ or PerCP-conjugated anti-mouse CD11b (clone M1/70) to determine leukocyte sub-types using a FACS Canto II flow cytometer. Antibodies were from BD Biosciences (San Jose, CA) and eBioscience (San Diego, CA).

### Leukocyte Functional Responses

#### Normal – Human Dermal Fibroblast phagocytosis and efferocytosis

Normal - Human Adult Dermal Fibroblasts (NHDF-Ad, Lonza) were thawed and grown in FBM growth medium (0.1% insulin, 0.1% rhFGF-B, 0.1% GA-1000, 2% FBS) for 3 days until 80–100% confluent. Cells were used within four healthy passages for phagocytosis assays. NHDF-Ad were plated into 96-well plates (Costar) at 5 × 10^3^ cells/well and cultured overnight. To assess phagocytosis, NHDF-Ad were pretreated with RvD3, RvD4 or vehicle (0.1–10 nM for 15 min at 37 °C) followed by incubation of fluorescent-labeled *S. aureus* (50:1) for 60 min. Phagocytosis of fluorescent-labeled *S. aureus* was assessed using an M3 SpectraMax plate reader at λ _exc_\λ _em_ - 480\516. Neutrophils for efferocytosis were isolated from human whole blood (obtained from consenting healthy human volunteers under protocol #1999-P001297 approved by Partners Human Research Committee) by density separation using Ficoll-Histopaque 1077-1. Cells were centrifuged (300 *g* for 30 minutes at 4 °C), and red blood cells were lysed to afford pure PMN. Freshly isolated PMN were stained with fluorescent nuclear dye bisbenzimide (Hoechst 33342; Sigma Aldrich; 1X concentration for 30 minutes at 37 °C) and cultured overnight (3 × 10^6^ cells/well in DPBS^+/+^). Before assessing efferocytosis, NHDF were pretreated with either RvD4 (0.1–10 nM), RvD1 (1 nM), RvD2 (1 nM), RvD3 (1 nM), MaR1 (1nM) or vehicle for 15 min at 37 °C. Fluorescent-labeled apoptotic PMN (3:1) were incubated for 60 min and efferocytosis was assessed using an M3 SpectraMax plate reader at 346, 460 nm respectively.

#### Human macrophage phagocytosis and efferocytosis

Human macrophages were prepared from peripheral blood mononuclear cells from the Children’s Hospital Boston Blood Bank and isolated by density-gradient Ficoll-Histopaque isolation. These were differentiated and cultured for 7 days in RPMI 1640 (10% fetal bovine serum) and GM-CSF (20 ng/mL). Macrophages were plated into 96-well plates (Costar) at 5 × 10^4^ cells/well the previous night and were pretreated with either RvD3, RvD4 or vehicle (0.1–10 nM for 15 min at 37 °C) followed by incubation of FITC labeled opsonized zymosan at 37 °C for 60 min or fluorescent-labeled *S. aureus* (50:1) for 60 min and assessed using an M3 SpectraMax plate reader at λ _exc_\λ _em_ - 493/525 and 480\516, respectively. Efferocytosis was assessed by pretreating macrophages with RvD3, RvD4 (0.1–10 nM) or vehicle for 15 min at 37 °C. Fluorescent-labeled apoptotic PMN (3:1) were incubated for 60 min and efferocytosis was assessed using an M3 SpectraMax plate reader at λ _exc_\λ _em_ - 346, 460 nm, respectively.

### Targeted Lipid Mediator Profiling

All samples and enzyme incubations were stopped with two volumes methanol and by solid-phase extraction on C18 SPE columns (Waters). 500 pg of a deuterated standard mixture containing, d_4_-LTB_4_, d_4_-LXA_4_, d_4_-PGE_2_, d_8_-5S-HETE and d_5_-RvD2 was added to determine recovery and quantification. Mediators were analyzed using LC-MS-MS analysis by a Qtrap 6500 (ABSciex) equipped with an Agilent HP1100 binary pump and diode-array detector on a Agilent Poroshell 120 EC-C18 column (4.6 mm × 100 mm × 2.7 μm) with a gradient of methanol/water/acetic acid of 55:45:0.01 (v/v/v) to 100:0:0.01 at 0.5 mL/min flow rate. To identify and quantitate SPMs, we used multiple reaction monitoring (MRM) at the transition ion pair (375 > 101) with signature ion fragments specific for RvD4 (at least 6 diagnostic ions and calibration curves).

### Statistics

Results are expressed as means ± SEM. Comparison between two groups was analyzed using Student’s t test and between multiple groups using ANOVA. Statistical significance was considered to be *P* < 0.05.

## Additional Information

**How to cite this article**: Winkler, J. W. *et al.* Resolvin D4 stereoassignment and its novel actions in host protection and bacterial clearance. *Sci. Rep.* **6**, 18972; doi: 10.1038/srep18972 (2016).

## Figures and Tables

**Table 1:** Endogenous production of RvD1–4.

			*Tissue/Sample*					
			Human Plasma *(1950s NIST)*	Human Serum	Human Breast Milk	Murine Brain	Murine Spleen	Sardines *(clupea harengus)*
D-Series metabolome								
	Q1	Q3	(pg/ml)	(pg/ml)	(pg/ml)	(pg/mg)	(pg/mg)	(pg/mg)
RvD1	375	233	2.5 ± 0.2	31.7 ± 3.8	147.0 ± 47.2	0.1 ± 0.02	8.5 ± 1.6	0.05 ± 0.01
RvD2	375	215	*	11.7 ± 0.8	82.4 ± 28.0	*	1.0 ± 0.2	0.12 ± 0.01
RvD3	375	147	*	6.3 ± 3.2	7.2 ± 2.7	*	0.35 ± 0.1	*
**RvD4**	**375**	**101**	**3.5** ± **0.2**	**27.0** ± **9.8**	**27.4** ± **7.5**	**0.2** ± **0.02**	**6.8** ± **1.0**	**0.12** ± **0.03**

Endogenous levels of resolvins in mouse tissue, human serum, plasma and sardines using LM metabololipidomics. Results are mean ± SEM, n = 3 exudates. Q1 (parent ion) and Q3 (diagnostic daughter ion).

asterisk (*) = below limit, limit ~ 0.1 pg in matrix
